# Quercetin ameliorates nicotine-induced spermatogenesis damage via modulation of sperm miR-151a-5p and testicular Cep72 gene expression

**DOI:** 10.1186/s12610-025-00288-9

**Published:** 2025-10-22

**Authors:** Masoumeh Faghani, Mahmoud Alijani, Aghil Esmaeili-bandboni, Fahimeh Mohammadghasemi

**Affiliations:** 1 Department of Anatomical Sciences, School of Medicine, Guilan University of Medical Sciences, Rasht, Iran; 2 Cellular and Molecular Research Center, School of Medicine, Guilan University of Medical Sciences, Rasht, Iran; 3 Department of Medical Biotechnology, School of Paramedical Science, Guilan University of Medical Sciences, Rasht, Iran

**Keywords:** Quercetin, Nicotine, Testis, Cep72, miR-151a-5p, Quercétine, Nicotine, Testicules, Cep72, miR-151a-5p

## Abstract

**Background:**

Smoking impairs spermatogenesis by disrupting gene and miRNA expression profiles. This study aims to explore the protective effects of quercetin against nicotine-induced testicular damage, with a specific focus on its regulatory role on sperm miR-151a-5p and testicular Cep72 gene expression, as potential molecular mechanisms involved in male reproductive dysfunction.

Male BALB/c mice (*N* = 32) were randomly divided into four groups of: control, nicotine, quercetin, and quercetin + nicotine combined groups. Treatments lasted 35 days. Spermatogenesis was evaluated through histopathological studies of the testicles. TAC and SOD levels were evaluated as markers of antioxidant activity using colorimetric methods in testicular homogenates. Sex hormones were measured using the ELISA method. Relative expression of Cep72 and miR-151a-5p genes in testicular tissue and epididymal sperm was assessed by real-time PCR. Androgen receptor (AR) and estrogen receptor alpha (ERα) expression in testicular tissue were evaluated by immunohistochemical methods.

**Results:**

The combined treatment of quercetin and nicotine enhanced sperm quality, caused significant changes in LH and estradiol hormone levels, increased anti-oxidants in testicular homogenates, and increased AR expression in Sertoli cells without affecting ERα, compared to the nicotine group. In addition, the combined therapy improved spermatogenesis by increasing the number of germ cells and Leydig cells. Furthermore, combined therapy increased testicular Cep72 gene expression and reduced sperm miR-151a-5p.

**Conclusions:**

This study demonstrates that quercetin may protect against nicotine-induced testicular damage by maintaining hormonal balance, enhancing AR expression, improving antioxidant enzyme activity, and normalizing the miR-151a-5p/Cep72 regulatory axis, ultimately supporting sperm quality and spermatogenesis. These results establish a foundation for future mechanistic and translational research.

## Résumé

**Contexte:**

Le tabagisme altère la spermatogenèse en perturbant les profils d’expression des gènes et des miARN. Cette étude vise à explorer les effets protecteurs de la quercétine contre les lésions testiculaires induites par la nicotine, en mettant l’accent sur son rôle régulateur sur l’expression du gène miR-151a-5p des spermatozoïdes et du gène Cep72 testiculaires, en tant que mécanismes moléculaires potentiels impliqués dans le dysfonctionnement reproductif masculin. Des souris mâles BALB/c (*n* = 32) ont été réparties au hasard en quatre groupes: groupe témoin, groupe nicotine, groupe quercétine et groupe combiné quercétine + nicotine. Les traitements ont duré 35 jours. La spermatogenèse a été évaluée par des études histopathologiques des testicules. Les niveaux de TAC et de SOD ont été évalués en tant que marqueurs de l’activité antioxydante à l’aide de méthodes colorimétriques dans les homogénats testiculaires. Les hormones sexuelles ont été mesurées par ELISA. L’expression relative des gènes Cep72 et miR-151a-5p dans le tissu testiculaire et les spermatozoïdes épididymaires a été évaluée par PCR en temps réel. L’expression des récepteurs aux androgènes (RA) et des récepteurs alpha aux œstrogènes (REα) dans le tissu testiculaire a été évaluée par méthodes immunohistochimiques.

**Résultats:**

Le traitement combiné quercétine + nicotine a amélioré la qualité des spermatozoïdes, provoqué des changements significatifs dans les niveaux de LH et d’œstradiol, augmenté les antioxydants dans les homogénats testiculaires et augmenté l’expression du RA dans les cellules de Sertoli sans affecter REα, par rapport au groupe nicotine. De plus, la thérapie combinée a amélioré la spermatogenèse en augmentant le nombre de cellules germinales et de cellules de Leydig. En outre, la thérapie combinée a augmenté l’expression du gène Cep72 testiculaire et réduit le miR-151a-5p des spermatozoïdes.

**Conclusions:**

Cette étude montre que la quercétine peut protéger contre les dommages testiculaires induits par la nicotine en maintenant l’équilibre hormonal, en améliorant l’expression du RA, en améliorant l’activité enzymatique antioxydante et en normalisant l’axe régulateur miR-151a-5p/Cep72, favorisant ainsi la qualité des spermatozoïdes et la spermatogenèse. Ces résultats jettent les bases de futures recherches mécanistes et translationnelles.

## Introduction

Nicotine, a toxic component of cigarette smoke, exerts adverse effects on various organs, including the reproductive systems of both males and females [1–3]. It can impair spermatogenesis and male fertility via multiple mechanisms, including oxidative stress, lipid peroxidation, endocrine hormone imbalance, inflammation, DNA fragmentation, and apoptosis [4]. Nicotine’s effect on reactive oxygen species (ROS) plays a key role in disrupting male reproductive function [5, 6]. Elevated ROS levels in the testes reduce sperm count, motility, maturation, and viability by altering mitochondrial membrane potential, inducing changes in sperm DNA methylation and hydroxymethylation, and triggering apoptosis [4, 7].

Lifestyle and diet influence the expression and regulation of epigenetic mechanisms, including sperm DNA methylation, histone modifications, and small non-coding RNAs (sncRNAs) [8]. Smoking and nicotine may affect the individual’s epigenetic profile through DNA methylation, altered gene transcription, and dysregulation of sncRNAs, such as miRNAs [8, 9]. Epigenetic features regulate gene expression at various stages of sperm development [10]. MicroRNAs exert negative regulation of gene expression by interacting directly with mRNA transcripts and modulating post-transcriptional processes [11, 12]. miRNAs are present in various cell types and tissues, including mature sperm, testes, epididymis, and prostate [13]. Current evidence suggests that cigarette smoking, advanced age, endocrine disruption, ROS, and environmental pollutants influence DNA methylation, miRNA dysregulation in sperm cells, and germ cell proliferation [10, 11, 14]. It has been shown that the upregulation and downregulation of specific miRNAs such as miR-151a-5p, miR-222-5p, miR-145-5p, miR-122-5p, and miR-1973 play distinct roles in spermatid formation, spermatogonial proliferation, and spermatogenesis [12, 15]. Reduced plasma levels of miR-151a-3p have been associated with heavy smoking and may indicate an elevated risk of intracranial aneurysm rupture in smokers [16]. Zhou et al. demonstrated a potential role for increased seminal expression of miR-151a-3p, particularly in relation to mitochondrial function, in the pathogenesis of asthenozoospermia [17]. Studies have shown that profiling miRNA expression patterns in sperm can serve as a biomarker for assessing sperm maturation and functional competence [15]. Cep72, a member of the centrosomal protein (Cep) family and a component of pericentriolar material, plays a critical role in mitosis in somatic cells. Alterations in Cep72 levels—via either overexpression or knockdown—disrupt key mitotic processes, including spindle assembly, microtubule organization, and accurate chromosomal alignment [18, 19]. Cep72 is predominantly expressed in pro-meiotic spermatocytes (Leptotene to Metaphase II; Lep–MII) and post-meiotic spermatids (Round Spermatids 2–6; RS2–RS6) within the mouse testis, although its expression is also detected in several other tissues [18]. Studies have shown that Cep72 knockout in mice leads to abnormal morphology of the sperm head and flagellum, resulting in increased numbers of morphologically defective sperm and reduced sperm motility [18, 20]. Prenatal exposure to environmental tobacco smoke has been shown to reduce the expression of Cep250 and centromere-associated protein E in the developing mouse hippocampus, both of which are involved in regulating cell proliferation [21].

There is substantial evidence that antioxidant agents, by reducing oxidative stress, can modulate hormonal balance, epigenetic regulation, and sncRNA expression, thereby influencing spermatogenesis [4, 7, 15]. Phytoestrogens, which act as antioxidants and structural analogs of estrogen, influence sncRNA expression and epigenetic regulation by reducing oxidative stress [22]. Furthermore, several studies have demonstrated that flavonoids can modulate both miRNA and gene expression [23, 24]. Quercetin is a naturally occurring phytoestrogen abundant in colorful vegetables, fruits, and flowers [25]. Studies have shown that quercetin, due to its antioxidant and flavonoid properties, can reduce ROS levels and may protect the male reproductive system [26, 27].Quercetin promotes healing in various diseases by altering microRNA levels, as shown in a 2021 study where it adjusted specific microRNAs in the brains of Alzheimer’s disease model mice with vitamin D deficiency [28]. Specifically, quercetin differentially regulates microRNA expression, increasing miR-146a in breast cancer cells and decreasing miR-21 in prostate cancer cells [29].

The aim of this study is to investigate the protective mechanisms of quercetin in alleviating nicotine-induced spermatogenic damage, specifically through the modulation of sperm miR-151a-5p and testicular Cep72 gene expression. This research seeks to elucidate the roles of these molecules in nicotine-related testicular dysfunction and to clarify how quercetin enhances sperm parameters and testicular function.

## Materials and methods

### Ethical statements

All experimental procedures were reviewed and approved by the Institutional Animal Care and Use Committee (GUMS) of Guilan University of Medical Sciences, under ethics approval number IR.GUMS.AEC.1402.013.

### Animal model

In this experimental study, 32 male BALB/c mice, aged 8 to 10 weeks and weighing between 20 and 25 g, were randomly obtained from the animal house of the School of Pharmacy, Guilan University of Medical Sciences. The mice were acclimatized for one week in Plexiglas cages, with unrestricted access to food and water, and maintained under a 12-h light/dark cycle. They were then divided into four groups:

A. Control Group: Received normal saline (0.9% NaCl) via intraperitoneal (IP) injection.
B. Nicotine Group: Received nicotine at a dosage of 0.6 mg/kg via intraperitoneal injection. The nicotine dose chosen for this study (0.6 mg/kg) corresponds to the serum nicotine levels found in individuals who smoke 12 cigarettes daily [30].
C. Quercetin Group: Received quercetin at a dosage of 15 mg/kg, dissolved in 0.3% ethanol, administered orally [31]. The quercetin dose used in this study was determined based on earlier research conducted on the testis function [31] and our initial laboratory investigations.
D. Nicotine + Quercetin Group: Received nicotine at 0.6 mg/kg via intraperitoneal injection and quercetin at 15 mg/kg via oral administration.

All treatments were administered once daily for a duration of 35 consecutive days. Nicotine (Sigma-Aldrich, Cat.no: N3876) was diluted with 0.9% normal saline for injection, while quercetin powder (Sigma-Aldrich, Cat. No: Q4951) was prepared in a 0.3% ethanol solution at the required concentration. After 35 days of treatment, the animals were anesthetized intraperitoneally using a mixture of 0.1 ml/100 g body weight containing ketamine (100 mg/ml) and xylazine (20 mg/ml). Blood, epididymis, and testicular samples were then collected. The testes were excised under sterile conditions via appropriate surgical incisions.

The left testis from each mouse was fixed in labeled containers with 10% neutral-buffered formalin for at least 72 h for histological and immunohistochemical analysis. The right testis was divided into two halves: one half was used to assess Cep72 gene expression via quantitative real-time PCR, while the other half was reserved for the analysis of stress markers. Both samples were transferred into separates crayotubes and were reserved in nitrogen tank. Additionally, the epididymal tail was collected both for evaluating sperm parameters and for extracting sperm to study the expression of miR-151a-5p.

### Sex hormones analysis

Blood samples were collected from the inferior vena cava of each animal under sterile conditions. The samples were centrifuged at 4,000 rpm for 20 min to isolate the serum, followed by an additional microcentrifugation at the same speed for 10 min. The isolated serum was stored in microtubes at −20 °C until subsequent biochemical analyses.

Serum levels of estradiol, testosterone, and luteinizing hormone (LH) were measured using the enzyme-linked immunosorbent assay (ELISA) technique, in accordance with the protocols provided by the respective kits.

For the measurement of testosterone, estradiol, and LH, diagnostic kits from Monobind, USA were used. (Cat. No: 3725-300A for testosterone, Cat. No: 4925-300A for estradiol, and Cat. No: 625-300A for LH. Absorbance was recorded at a wavelength of 450 nm. The sensitivities of the assays were 8.2 pg/mL for estradiol, 0.057 ng/mL for testosterone, and 0.010 mIU/mL for LH, respectively.

### Histology and spermatogenesis evaluation

After fixation, the testicular tissue samples were processed using a tissue processor. The samples were dehydrated in ascending concentrations of ethanol, followed by infiltration with xylene and molten paraffin, which was also used for embedding the tissues. Sections were cut at a thickness of 5 μm, spaced at 50 μm intervals, using a rotary microtome (Zeiss-Germany). Three sections were prepared from each animal.

Slides were stained with hematoxylin and eosin (H&E) and examined under an Olympus light microscope. Spermatogenesis was evaluated qualitatively and quantitatively.

Seminiferous tubules at stages VII–VIII were identified, and photomicrographs were captured and analyzed using Digimizer image analysis software. Within each slide, counts of spermatogonia, primary spermatocytes, round spermatids, Sertoli cells, and Leydig cells were performed in a designated area of 2,500 μm^2^. Since spermatogenesis in mice comprises 12 stages, and secondary spermatocytes are predominantly found at stage XII, only stage XII seminiferous tubules were analyzed for their presence [32].

To assess the maturity and quality of spermatogenesis in the seminiferous tubules, the Johnsen scoring system was utilized. For this purpose, 20 seminiferous tubules were selected from each animal and scored on a scale from 1 to 10 based on the score criteria and morphological features of the tubules. Each cross-section was evaluated as follows [33].

- 10: Complete spermatogenesis, with numerous sperm heads located at the lumen’s periphery
- 9: A large number of sperm present, but the lumen is irregular and not round
- 8: Very few sperm are present
- 7: No sperm visible, but numerous round spermatids are present
- 6: A few round spermatids are observed
- 5: No sperm or round spermatids are visible, but many primary spermatocytes are present
- 4: Very few primary spermatocytes are visible
- 3: No primary spermatocytes are observed; only spermatogonia are present
- 2: No germ cells are visible; only Sertoli cells are present
- 1: Neither germ cells nor Sertoli cells are visible, and the tubules are atrophic

### Expression of androgen receptors and estrogen alpha receptors in the testis

To evaluate androgen receptors (AR) and estrogen receptor alpha (ERα) in the testes, immunohistochemistry (IHC) was performed. Primary antibodies were obtained from Zytomed, Germany (AR, Cat. No: PDM 167) and Master, Spain (ERα, Cat. No: MAD-000306QD-7). All procedures were carried out according to the manufacturers’ instructions. Paraffin-embedded tissue samples were sectioned at 3 μm thickness on poly-L-lysine-coated slides, followed by deparaffinization, rehydration, and endogenous peroxidase blocking. Antigen retrieval was performed by microwave heating in Tris–EDTA buffer (pH 9.0) for 40 min. The sections were then washed with PBS and incubated with primary monoclonal rabbit antibodies against AR or ERα for 90 min at room temperature. Subsequently, a polymer-based enhancer was applied for 30 min at room temperature. After three washes with PBS, horseradish peroxidase (HRP; Zytomed, Germany, Cat. No: HRP-060) was used as the enzymatic label for 15 min at room temperature. Visualization was achieved with the chromogen diaminobenzidine (DAB), followed by counterstaining with Mayer’s hematoxylin. Stained sections were examined under an Olympus light microscope, with brown staining indicating positive expression of AR or ERα.

For each animal, one slide was analyzed. Between 15 and 20 seminiferous tubules at stages VII–VIII were selected, and brown-stained Sertoli cells, Leydig cells, and germ cells were counted to calculate average expression levels. All cell counts were performed using Digimizer software within a defined area of 2,500 μm^2^.

### Evaluation of epididymal sperm parameters

To evaluate sperm count, motility, and morphological abnormalities, samples were collected from the epididymal tail. The tissue was placed in a dish containing Ham’s F-10 medium, pre-warmed in an incubator at 37 °C. The tissue was gently minced with a scalpel to release the spermatozoa, and the suspension was allowed to settle for at least 20 min at 37 °C. Sperm counts were performed using a Neubauer hemocytometer, and motility and morphological abnormalities were assessed according to previously published protocols [34].

### Evaluation of stress markers in testicular tissue homogenate

A portion of the right testicular tissue was promptly snap-frozen in liquid nitrogen and stored at − 80 °C for subsequent analysis of oxidative stress and antioxidant markers. For biochemical assays, the frozen tissues were thawed, weighed, and homogenized in ice-cold phosphate-buffered saline (PBS) for 5 min, followed by centrifugation at 13,000 × g for 10 min at 4 °C. The resulting supernatants were collected and stored at − 80 °C until further analysis.

Antioxidant enzyme activities, including superoxide dismutase (SOD) and total antioxidant capacity (TAC), were measured using commercial colorimetric kits (Cat. No: KZSO-OB, Kushan Zist, Iran; and Cat. No: KZTA-OB, Teb Pazhouhan Razi, Iran) at 450 nm. The detection limits of the assays were 0.1 U/mL for SOD and 0.5 U/mL for TAC. Total protein content was determined using the Bradford assay (Cat. No: KZBF-OX, Kushan Zist, Iran) following the manufacturer’s instructions and previously described protocol [2, 35], to ensure accurate normalization of the biochemical data.

### Isolation of sperm for evaluation of miR-151a-5p

To detect miR-151a-5p in epididymal sperm, spermatozoa were isolated as follows: the caudal part of the epididymis was bilaterally removed and placed in a dish containing 1 mL of pre-warmed (37 °C) Ham’s F-10 Nutrient Mix (Cat. No: 11550043, Gibson; Thermo Fisher Scientific, Scotland). The tissue was finely minced using forceps and incubated at 37 °C for 20 min to allow mature sperm to swim out. Larger tissue fragments were discarded. The remaining steps were performed in a warm room maintained at 37 °C. The sperm-containing medium was centrifuged at 3,000 rpm for 8 min, after which the supernatant was removed. Next, 400 μL of fresh, warmed Ham’s F-10 medium was gently added to the pellet. The tubes were positioned at a 45° angle and incubated for 45 min to allow motile sperm to swim up into the fresh medium. The supernatant, now enriched with motile sperm, was collected and centrifuged again at 3,000 rpm for 8 min to pellet the sperm. The supernatant was discarded, and finally, 1,000 μL of TRIzol® reagent was added. The mixture was gently pipetted to ensure complete homogenization, then stored at − 80 °C until further use [36].

### RNA extraction for quantitative real-time PCR (qPCR)

To evaluate the expression levels of the Cep72 gene and miR-151a-5p in testicular tissue and epididymal sperm samples, total RNA was extracted using RNX-Plus reagent (Cat. No: EX6101; SinaClone, Iran) according to the manufacturer’s instructions. The concentration, purity, and integrity of the extracted RNA were assessed by measuring the optical density at 260/280 nm with a NanoDrop spectrophotometer. RNA integrity was further verified by electrophoresis on a 2% agarose gel.

### Primer design for Cep72 and miR-151a-5p

Primers for the amplification of Cep72 and GAPDH (used as an internal control) were designed using the NCBI Primer-BLAST tool (https://www.ncbi.nlm.nih.gov/tools/primer-blast). For the detection of miR-151a-5p, the stem-loop reverse transcription quantitative PCR (RT-qPCR) method was employed. This technique is highly sensitive and specific for detecting and quantifying small RNAs, particularly mature microRNAs.

The method uses a stem-loop RT primer that consists of a short 3′ overhang, a stem, and a loop (typically 5–8 nucleotides). This structure allows the primer to bind specifically to the 3′ end of the target miRNA, thereby enhancing both the sensitivity and specificity of detection compared to conventional qPCR approaches.

The mature sequence of miR-151a-5p was obtained from the miRBase database. The stem-loop RT primer, along with the forward and reverse primers, were designed based on established protocols and previously reported sequences in the literature [37–39]. U6 small nuclear RNA (snRNA) was used as the internal control for miR-151a-5p expression analysis. All primers were synthesized by SinaClon Co., Iran. The sequences are listed in Table 1.

**Table 1:** Sequences of primers used in the study

Primer name	Sequence (5´ → 3´)	Tm(°C)
Stem loop RT-primer	GAAAGAAGGCGAGGAGCAGATCGAGGAAGAAGACGGAAGAATGTGCGTCTCGCCTTCTTTCACTAGACT	––-
Reverse primer for miR-151-5p	GCGAGCACAGAATTAATACGAC	58.90
Forward primer for miR-151-5p	GGTCGAGGAGCTCACAGTCT	59.63
Forward primer for U6snRNA	CTCGCTTCGGCAGCACA	60.42
Reverse primer for U6snRNA	AACGCTTCACGAATTTGCGT	59.69
Forward primer for Cep72	CCGGCGTCGAGTTTGAAAATA	59.81
Reverse primer for Cep72	GATAAAGATCGAAGCTCAGCCAG	59.46
Forward primer for GAPDH	CTCTCTGCTCCTCCCTGTTC	59.18
Reverse primer for GAPDH	ACCGACCTTCACCATCTTGT	58.94

Gene expression levels were analyzed using the ${2}^{-\Delta \Delta CT}$ method. In this study, data from each experimental group were compared to the average values of the control group. GAPDH and U6snRNA were used as internal reference genes for the normalization of mRNA and miRNA expression, respectively.

### cDNA synthesis and quantitative real-time PCR procedure

Following RNA extraction, reverse transcription was performed using the First Strand cDNA Synthesis Kit (Cat. No: RT5201; SinaClon, Iran). For cDNA synthesis, the reaction mixture (final volume: 20 µL) consisted of 7 µL RNA, 5 µL stem-loop primer (for miRNA detection) or a mixture of oligo(dT) and random hexamer primers (for mRNA targets), 2 µL thermostable reverse transcriptase, 4 µL 5 × RT buffer, and 2 µL dNTPs.

Reverse transcription was carried out under the following thermal conditions: 25 °C for 10 min, 47 °C for 60 min, and 70 °C for 10 min, followed by immediate chilling on ice. The resulting cDNA was stored at –70 °C until further use.

Quantitative real-time PCR (qRT-PCR) was performed in 20 µL reaction volumes containing 2 µL cDNA, 3 µL primers, 5 µL double-distilled water, and 10 µL Real Plus 2 × Master Mix Green (Cat. No: A325402; Ampliqon, Denmark). Amplification was conducted on an ABI 7300 Real-Time PCR System (Applied Biosystems, USA) according to the manufacturer’s instructions.

### Statistical analysis

Statistical analyses were performed using GraphPad Prism version 10 (GraphPad Software Inc., USA). Data are presented as mean ± standard error of the mean (SEM). The normality of data distribution was tested using the D’Agostino–Pearson omnibus test, and homogeneity of variances was evaluated using the Brown–Forsythe test. Based on the results of these preliminary tests, either one-way analysis of variance (ANOVA) or the Kruskal–Wallis test was applied, depending on whether the data met the assumptions of normality and homoscedasticity. Statistical significance was considered at *P* < 0.05.

## Results

### Hormones

**Fig. 1:**
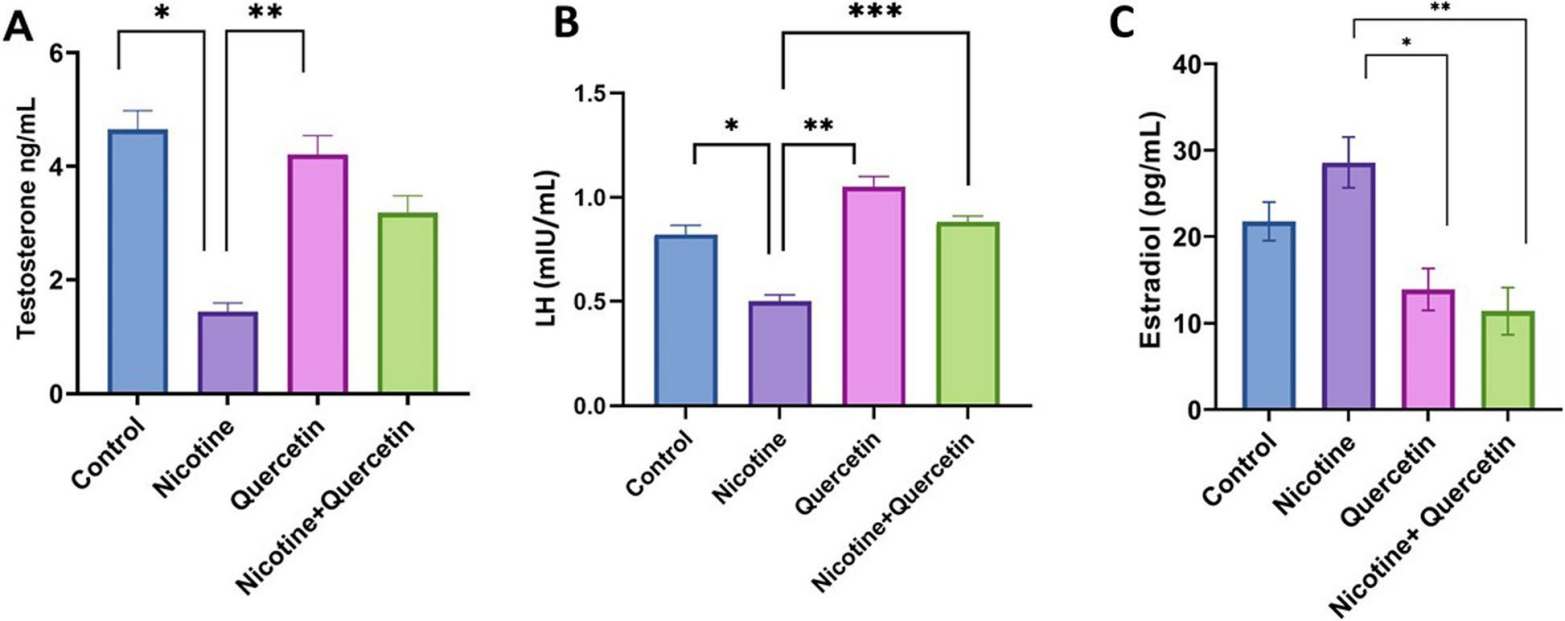
Serum hormone levels in different groups. **A** shows testosterone alterations. *p = 0.005, **p = 0.018. **B** Shows LH alterations. *p = 0.006, **p = 0.001, ***p = 0.005. **C** Shows estradiol alterations. *p = 0.005, **p = 0.001. Data are expressed as mean ± SE. *n* = 8 per group

Administration of nicotine significantly reduced serum testosterone (*p* = 0.005) and LH levels (*p* = 0.006) compared with the control group. Quercetin treatment alone led to an increase in both testosterone (*p* = 0.018) and LH (*p* = 0.001) compared with the nicotine (Fig. 1A and B). Moreover, treatment with quercetin markedly improved serum LH levels in the quercetin–nicotine group compared with the nicotine group (*p* = 0.005; Fig. 1). Although testosterone levels tended to increase following quercetin treatment, this effect did not reach statistical significance in the quercetin–nicotine group.

Serum estradiol levels were elevated in the nicotine group compared with the control, although this difference was not statistically significant. In contrast, estradiol levels were significantly reduced in both the quercetin group (*p* = 0.005) and the quercetin–nicotine group (*p* = 0.001) compared with the nicotine group. These findings suggest that co-treatment with quercetin exerts protective effects on LH and estradiol levels when compared with nicotine treatment alone (Fig. 1C).

### Epididymal sperm parameters

**Fig. 2:**
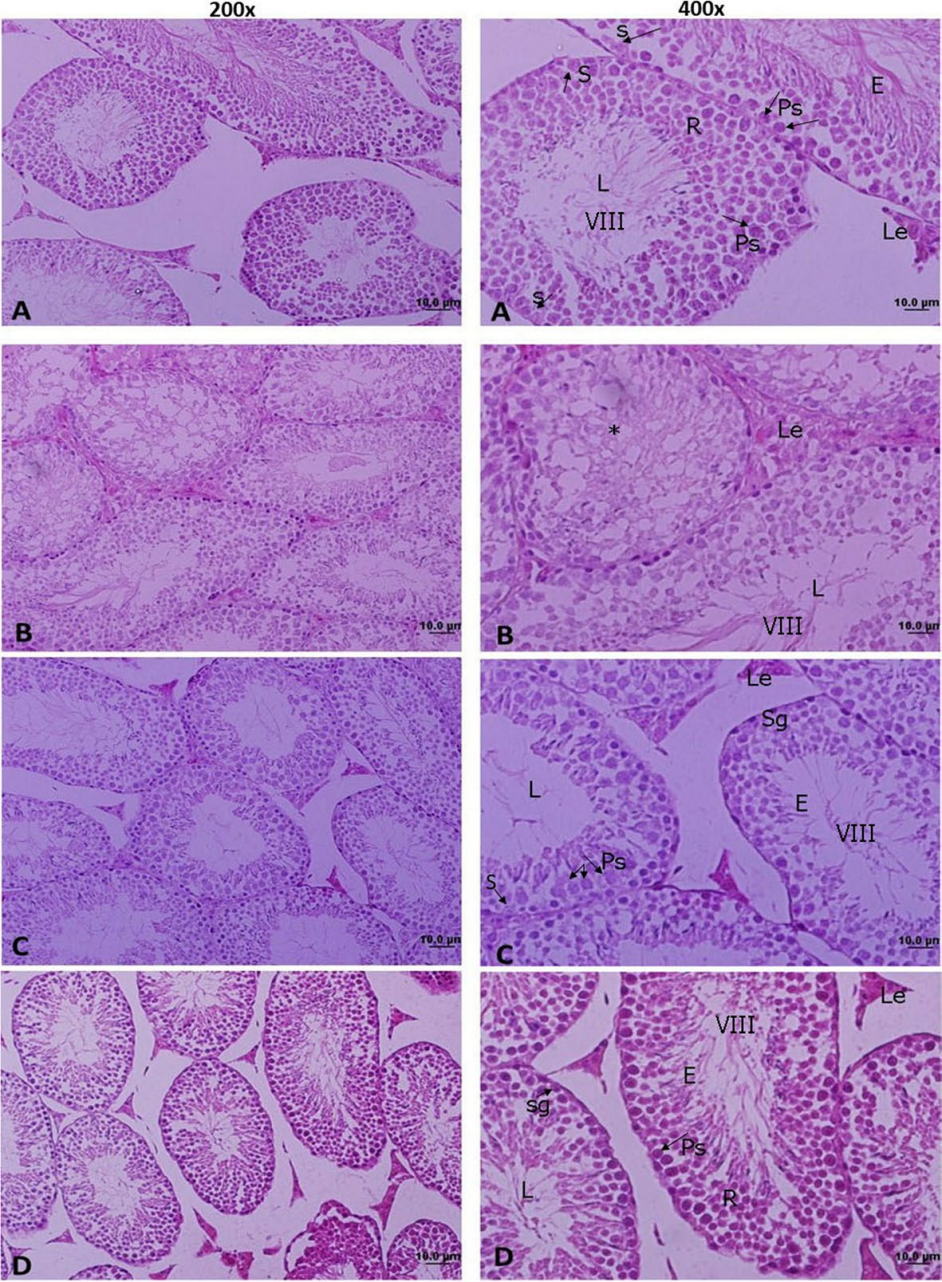
Light microscopic images of testicular tissue in mice: **A** control, **B** nicotine, **C** quercetin, and **D** nicotine–quercetin groups. L: lumen; VIII: stage VIII of spermatogenesis; S: Sertoli cell; Sg: spermatogonia cell; Ps: primary spermatocyte; R: round spermatid; E: elongated spermatid; Le: Leydig cell. Nicotine treatment markedly reduced the quality and quantity of seminiferous tubules. In some tubules (indicated with an asterisk *), the organization of spermatogenesis was altered. Co-treatment with nicotine and quercetin improved both the structural integrity and cellular composition of seminiferous tubules compared to nicotine alone. Hematoxylin and eosin staining; magnification 200 × and 400 ×

Table 2 presents the sperm parameters and spermatogenesis status across the experimental groups. Nicotine administration significantly decreased sperm count (*p* = 0.001), the percentage of morphologically normal sperm, and sperm motility compared with the control group (*p* = 0.001). Importantly, co-treatment with quercetin and nicotine significantly improved epididymal sperm parameters relative to the nicotine group (*p* = 0.001) (Table 2, Fig. 2).

**Table 2:** The effect of nicotine and quercetin on the sperm parameters, germ and somatic cells in adult male mice

Parameter	Control	Nicotine	Quercetin	Nicotine- quercetin
Sperm count × 10^6^	122.3 ± 10.95	56.86 ± 7.59^a^	113.8 ± 8.76^b^	102.4 ± 11.57^b^
Sperm motility %	71.69 ± 4.41	42.30 ± 3.6^a^	69.39 ± 4.58^b^	59.24 ± 3.79^b^
Sperm normal morphology %	37.01 ± 2.29	15.82 ± 3.48^a^	39.82 ± 3.74^b^	33.01 ± 2.91^b^
Johnson score	9.16 ± 0.07	7.44 ± 0.18^a^	9.01 ± 0.14	9 ± 0.09^b^
Spermatogonia (n/f)	7.7 ± 0.25	5.07 ± 0.31	7.2 ± 0.28	6.35 ± 0.24
Primary spermatocyte(n/f)	11.20 ± 2.08	7.64 ± 1.2^a^	11.77 ± 1.51^b^	9.6 ± 1.38
Secondary spermatocyte(n/f)	2.76 ± 0.09	1.8 ± 0.11^a^	2.82 ± 0.1^b^	2.02 ± 0.09^ab^
Round spermatid (n/f)	21 ± 1.53	13.38 ± 1.58^a^	20.8 ± 1.49^b^	17.98 ± 3.31^b^
Sertoli cell (n/f)	1.4 ± 0.2	1.22 ± 0.16	1.65 ± 0.18	1.28 ± 0.15
Leydig cell (n/f)	4.08 ± 0.18	2.66 ± 0.1^a^	4.31 ± 0.16^b^	3.75 ± 0.15^b^

All data are expressed as mean ± standard error (SE)

*n/f* number per microscopic field

^a^Statistically significant compared with the control group (*p* = 0.001)

^b^Statistically significant compared with the nicotine group (*p* = 0.001). *n* = 8 per group

### Testis histology and spermatogenesis process

Nicotine administration led to a significant decline in the Johnsen score compared with the control group, whereas quercetin, either alone or in combination with nicotine, substantially enhanced spermatogenesis quality (*p* = 0.001; Table 2).

Exposure to nicotine reduced spermatogonia numbers, though this decrease did not reach statistical significance. Quercetin treatment, whether administered alone or together with nicotine, resulted in higher spermatogonia counts relative to the nicotine group; however, these differences were likewise not statistically significant.

Nicotine markedly decreased the numbers of primary and secondary spermatocytes as well as round spermatids compared with controls (*p* = 0.001). Importantly, co-administration of quercetin with nicotine significantly restored the counts of secondary spermatocytes and round spermatids when compared with nicotine alone (*p* = 0.001).

No significant differences were observed in Sertoli cell numbers across the groups. By contrast, nicotine exposure significantly reduced Leydig cell counts compared with controls (*p* = 0.001), whereas quercetin treatment, administered either independently or in combination with nicotine, significantly elevated Leydig cell numbers relative to the nicotine group (*p* = 0.001) (Fig. 2, Table 2).

### Expression of androgen receptors in testis

**Fig. 3:**
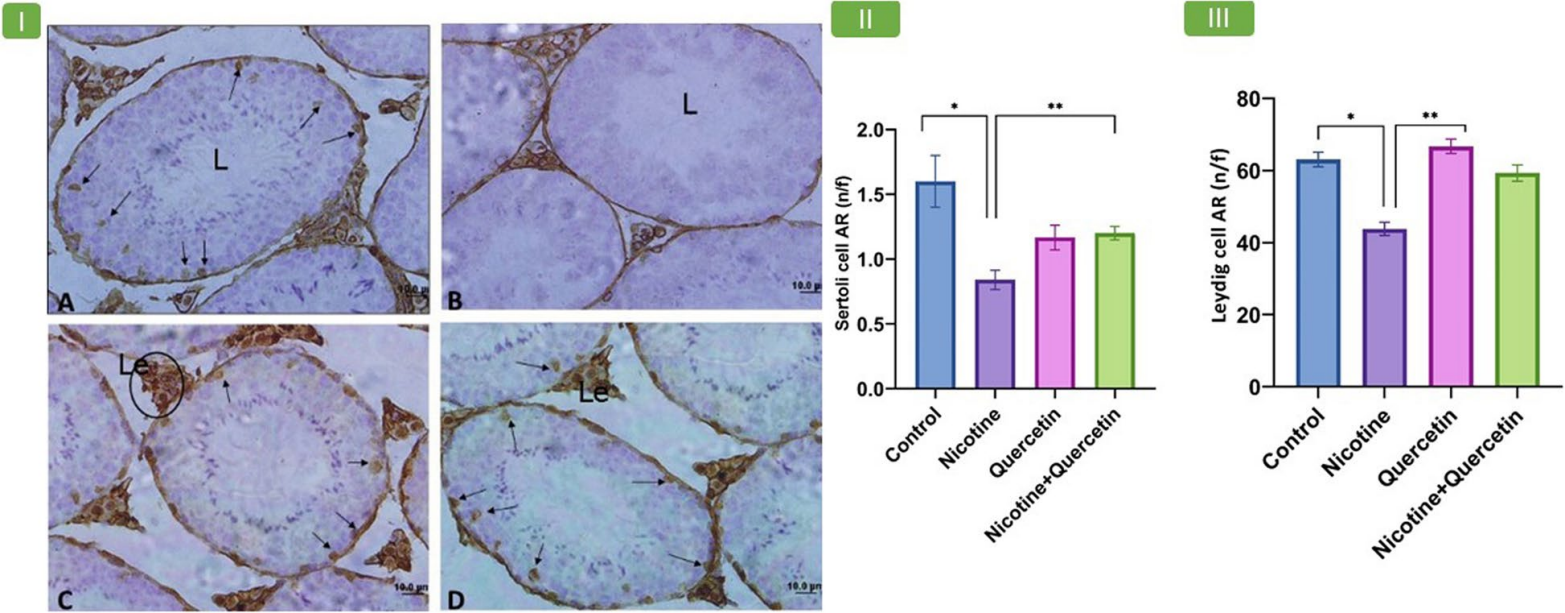
Panel 3-I shows photomicrographs of seminiferous tubules in mouse testis using immunohistochemical staining for AR across different experimental groups. (A) Control group, (B) Nicotine group, (C) Quercetin group, (D) Nicotine–quercetin group. Brown-stained cells represent those expressing the AR. Arrows within the seminiferous tubules indicate Sertoli cells, while black circles in the interstitial tissue denote Leydig cells (Le). L indicates the lumen. In both the control and quercetin groups, tear-shaped Sertoli cells within the seminiferous tubules show strong positive AR staining, while germ cells demonstrate minimal reactivity. In the interstitial tissue, Leydig cells with round, coarse nuclei also show positive AR expression. Magnification: 400 ×. Panel 3-II: shows Sertoli cell AR number/field (n/f). *p = 0.001, **p = 0.03. Panel 3-III: shows Leydig cell AR number/field (n/f). *p = 0.008, **p = 0.0002. Data are expressed as mean ± SE. *n* = 8 per group

Nicotine treatment significantly reduced AR expression in Sertoli cells compared with the control group (*p* = 0.001). Quercetin administration alone slightly increased AR expression compared with nicotine, but this change was not significant. However, co-treatment with nicotine and quercetin significantly improved AR expression compared with nicotine alone (*p* = 0.03) (Fig. 3I, II).

In Leydig cells, AR expression was significantly decreased following nicotine administration compared with the control group (*p* = 0.008). Quercetin treatment alone resulted in significantly higher AR expression than the nicotine group (*p* = 0.0002) (Fig. 3I, III).

### Expression of estrogen receptors (ERα) in testis

**Fig. 4:**
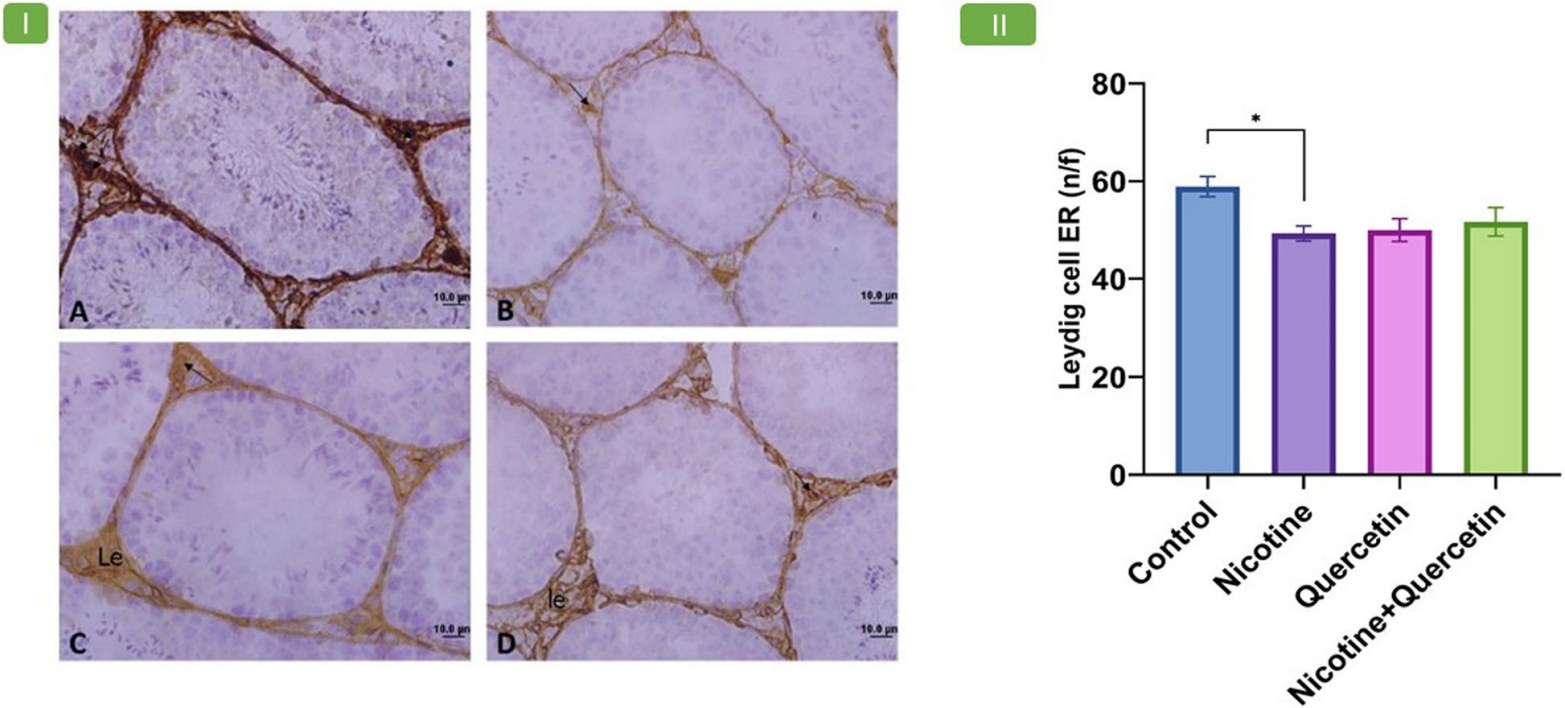
Panel 4-I shows photomicrographs showing seminiferous tubules of mouse testes stained by immunohistochemistry with an anti-estrogen receptor alpha (ERα) antibody in different experimental groups. (A) Control group; (B) Nicotine group; (C) Quercetin group; (D) Nicotine + Quercetin group. Brown-stained cells represent cells expressing ERα. In the interstitial tissue, Leydig cells are visible. Arrows indicate Leydig cells exhibiting a positive immunoreaction. Nicotine administration caused a significant reduction in the number of ERα-positive Leydig cells compared to the control group. Magnification: 400 ×. Panel 4-II: shows Leydig cell ERα number/field (n/f). *p = 0.03, Data are presented as mean ± SE (*n* = 8 per group)

Nicotine treatment significantly reduced ERα expression in Leydig cells compared with the control group (*p* = 0.03). However, no significant differences in ERα expression were observed between the quercetin and quercetin–nicotine groups relative to the nicotine group (Fig. 4I, II).

### Antioxidative markers

**Fig. 5:**
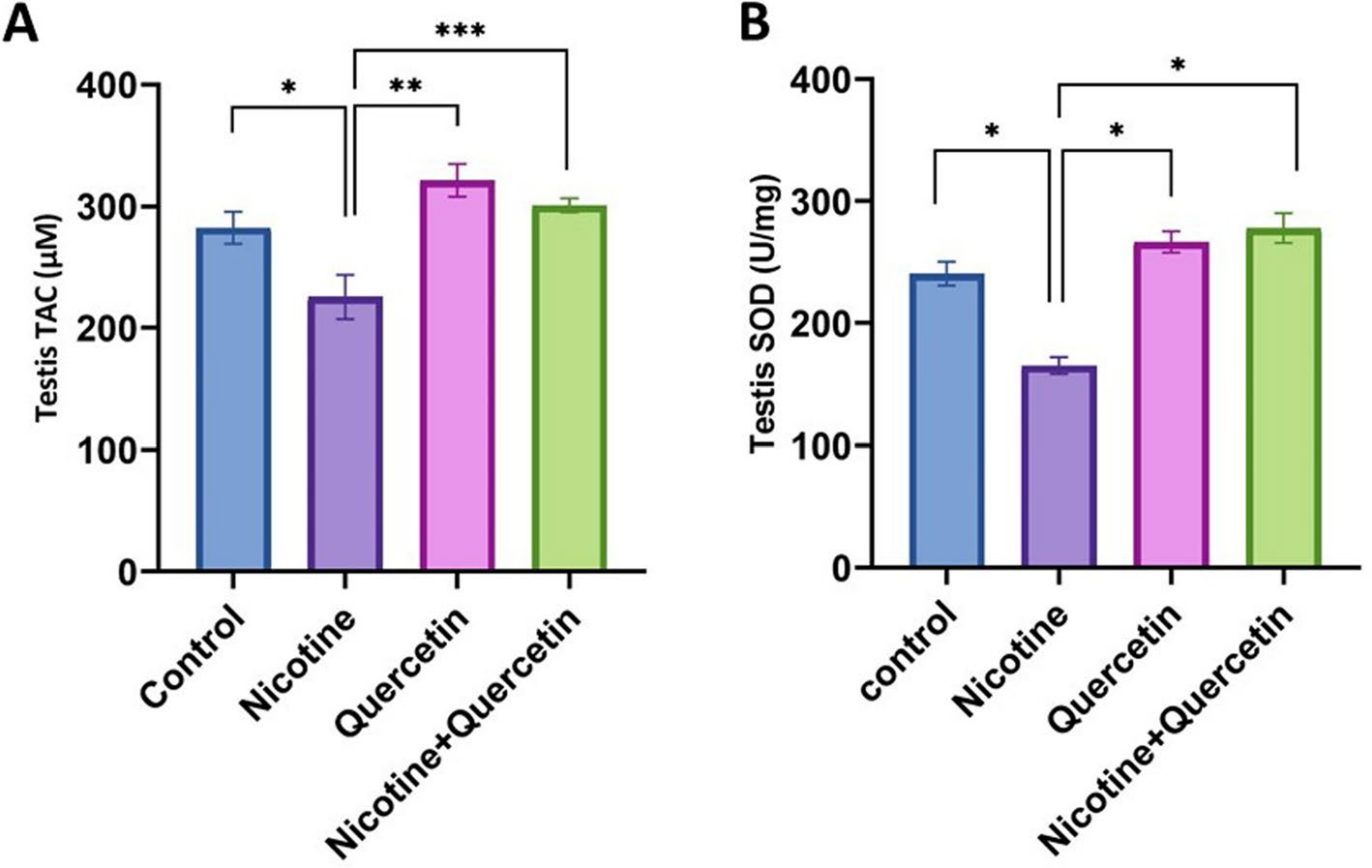
Antioxidant markers in testicular tissue homogenates of mice. **A** Shows total antioxidant capacity (TAC), *p = 0.03, **p = 0.0003, ***p = 0.004. **B** Shows superoxide dismutase (SOD) activity, *p = 0.0001. Data are presented as mean ± SE (*n* = 8 per group)

With respect to antioxidative markers, nicotine exposure resulted in a significant decline in SOD (*p* = 0.0001) and TAC (*p* = 0.03) activities compared with controls. In contrast, quercetin treatment markedly increased both SOD (*p* = 0.0001) and TAC (*p* = 0.0003) levels relative to the nicotine group. Similarly, co-administration of quercetin and nicotine significantly improved SOD (*p* = 0.0001) and TAC (*p* = 0.004) levels compared with nicotine treatment alone (Fig. 5A, B).

### Differential expression of miR-151a-5p and Cep72 in response to nicotine and quercetin

**Fig. 6:**
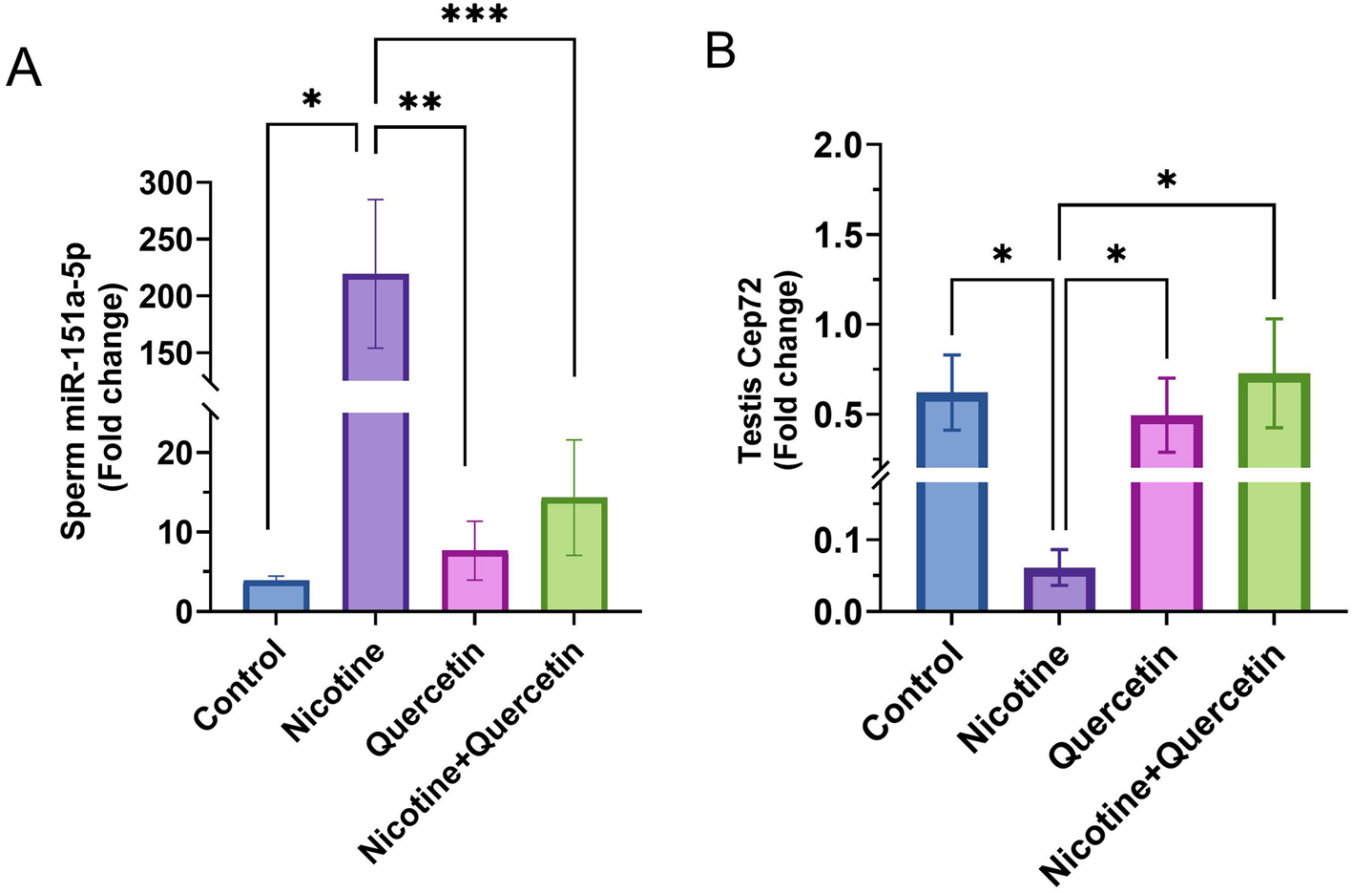
**A** Shows the expression level of miR-151a-5p in epididymal sperm, *p = 0.015, **p = 0.0004, ***p < 0.0009. **B** Shows the expression of Cep72 in testicular tissue, *p < 0.05, Data are expressed as mean ± SE. *n* = 8 per group

Nicotine treatment caused a significant up-regulation of miR-151a-5p expression compared to the control (*p* = 0.015). Quercetin administration markedly reduced its expression (*p* = 0.0004), and co-treatment with nicotine and quercetin also significantly down-regulated miR-151a-5p (*p* = 0.0009) (Fig. 6A).

Conversely, Cep72 expression was significantly down-regulated in the nicotine group compared with the control (*p* = 0.02). Quercetin treatment significantly restored Cep72 levels (*p* < 0.05), and co-treatment with nicotine and quercetin further increased expression toward near-control values (*p* < 0.05) Thus, quercetin may exert its protective role against nicotine toxicity by normalizing the miR-151a-5p/Cep72 regulatory axis (Fig. 6B).

## Discussion

In this study, we demonstrate that quercetin protects against nicotine-induced testicular injury by restoring hormonal balance, enhancing antioxidant activity, and upregulating androgen receptor expression. Importantly, our findings highlight a novel regulatory pathway involving miR-151a-5p and Cep72, suggesting a previously unrecognized mechanism through which nicotine disrupts spermatogenesis and quercetin provides protection. We observed a significant upregulation of miR-151a-5p in nicotine-exposed mice, accompanied by a marked downregulation of Cep72, a gene critically involved in centrosome function and sperm flagellar development. These changes resulted in reduced sperm quality and impaired spermatogenesis. Notably, aberrant overexpression of miR-151a-5p has also been implicated in pathological processes such as cancer and metabolic disorders [40]. miRNA profiles in sperm can indicate both sperm maturity and function [15]. In this study, nicotine increased miR-151a-5p expression in epididymal sperm, which was associated with impaired mitochondrial function and reduced sperm motility [41]. Smoking has been shown to alter sperm mRNA and miRNA levels, thereby affecting gene expression [42]. Endocrine disturbances and stress also influence sperm miRNA expression [43]. The observed suppression of Cep72 in the presence of elevated miR-151a-5p suggests a potential regulatory interaction that disrupts spermatogenic progression and compromises sperm quality. While several miRNAs, including miR-34c, miR-21, and miR-135a, have been linked to male fertility and spermatogenesis [41–43]. miR-151a-5p regulates various genes by binding to their mRNAs, and TargetScan analysis identifies Cep72 as one of its primary inhibitory targets. Spermatogenesis in mammals involves mitosis, meiosis, and spermiogenesis, with centrosomes playing a central role. Although the function of Cep72 in fertility is not yet fully understood, studies in mice lacking this gene show impaired sperm production, reduced sperm counts, and diminished fertility [22]. In the present study, nicotine decreased Cep72 expression in the testes. Given its role in mitosis, the cell cycle, and cell proliferation, reduced expression of Cep72 may contribute to the lower numbers of secondary spermatocytes and round spermatids observed in nicotine-treated mice. Supporting this, earlier research demonstrated that Cep72 is expressed in spermatocytes from the leptotene stage through metaphase II in mice [18]. Notably, quercetin treatment effectively reversed these alterations by reducing miR-151a-5p expression and partially restoring Cep72 levels toward baseline, thereby underscoring its modulatory effect on this novel regulatory axis.

Nicotine activates the hypothalamic–pituitary–adrenal (HPA) axis, raising corticosteroid levels that suppress GnRH and reduce LH secretion [44]. Lower LH, together with nicotine-induced downregulation of LH receptors, decreases testosterone production [45, 46]. Additional mechanisms contributing to reduced testosterone include oxidative stress, impaired testicular blood flow, disrupted lipid metabolism, and inhibition of steroidogenic enzymes [30, 35, 47]. In contrast, elevated LH observed in the nicotine–quercetin group may stimulate intratesticular signaling pathways (cAMP/PKA, steroidogenic enzymes, growth factors) and miRNA expression [43], thereby supporting germ cell maturation, centrosome stability, and flagellar assembly, which could explain the increased Cep72 expression.

The improvement in sperm parameters observed in the co-treated group likely stems from increased intratesticular testosterone (ITT) and hormonal modulation (higher LH, lower estradiol), rather than from detectable changes in serum testosterone. While ITT directly promotes spermatogenesis, serum testosterone may not always reflect local testicular steroidogenesis [45, 48]. However, the exact mechanism is un-known. Testosterone also supports chromatin remodeling [49], which may interact with Cep72 proteins.

Nicotine downregulates ERα [50] and elevates estradiol through altered metabolism or increased aromatase activity [48, 49], impairing sperm quality and fertility [51–53], partly via ER-mediated signaling and altered miRNA expression [51]. Elevated miR-100 and let-7b levels, along with reduced ERα expression, have been associated with increased risk and progression of infertility [52]. In contrast, quercetin may lower estradiol, thereby restoring testosterone levels [53]. Quercetin, a phytoestrogen, supports spermatogenesis by enhancing steroidogenesis through cAMP-dependent upregulation of genes such as StAR, Cyp11a1, and Fdx1, and by activating the transcription factor Creb1 [41]. Its antioxidant and anti-apoptotic effects protect Leydig cells, improving their function and sensitivity to LH, which in turn promotes testosterone secretion [54–56]. Thereby supporting germ cell maturation, centrosome stability, and flagellar assembly, which could explain increased Cep72 expression and sperm quality.

Androgen receptors (AR) are crucial for meiosis, spermiogenesis, and the release of mature spermatids [57]. In this study, nicotine reduced AR expression in Sertoli and Leydig cells [58, 59], likely due to oxidative stress and reduced Leydig cell numbers, leading to decreased testosterone production and impaired spermatid development [33, 60]. Nicotine also lowers serum LH and directly affects testicular cells through acetylcholine receptors, thereby disrupting steroidogenesis and AR signaling [5, 33, 35]. While phytoestrogens have shown mixed effects on AR [61, 62], antioxidants such as tocopherol may enhance AR expression under stress [63]. Although quercetin inhibits AR in prostate cancer cells [64], in this study, it restored the AR expression reduced by nicotine.

MicroRNAs such as miR-125b and miR-205 have been shown to regulate AR signaling in other contexts [65, 66]. Although there is no direct evidence that miR-151a-5p or Cep72 regulate AR expression in testicular tissue, the changes observed in their levels after nicotine and quercetin treatments indicate a possible association.

Bioinformatic analysis using the TargetScan database identified Cep72 mRNA as a predicted target of miR-151a-5p. An inverse correlation between miRNA and target gene expression, measured by real-time PCR (RT-qPCR), is widely accepted as initial evidence of miRNA regulation, as demonstrated in several studies [67, 68]. However, direct functional confirmation of miRNA targeting requires luciferase assays, which validate binding and repression of the target site [69]. Combining RT-qPCR expression data with luciferase assay results provides strong evidence for miRNA-mediated regulation [70].

Despite these promising findings, this study has several limitations.

First, although we identified an inverse relationship between miR-151a-5p and Cep72 expression, we did not perform luciferase reporter assays, which are considered the gold standard for validating direct miRNA–mRNA interactions.

Second, our work focused primarily on gene expression and antioxidant responses without including more detailed molecular endpoints such as protein phosphorylation, apoptotic markers, or mitochondrial function. Additionally, we did not include post-transcriptional steps in gene expression, which could have been evaluated using valuable techniques such as immunohistochemistry and Western blot. These methods could provide further insight into the protective mechanisms of quercetin.

Third, this study was performed in an animal model, which, while valuable, may not fully replicate the complexity of human reproductive physiology. Translation of these findings to clinical settings will require further studies in human samples or clinical trials.

In conclusion, the present study provides novel evidence that quercetin ameliorates nicotine-induced reproductive toxicity through modulation of miR-151a-5p and Cep72 expression, together with improvements in antioxidant defense, hormone balance, sperm quality, and spermatogenesis. These results establish a foundation for future mechanistic and translational research.

## Data Availability

The data that support the findings of this study are available from the corresponding author and author initials, upon reasonable request.

## Abbreviations

ROS: Reactive oxygen species
sncRNAs: Small non-coding RNAs
LH: Luteinizing hormone
ELISA: Enzyme-linked immunosorbent assay
AR: Androgen receptors
ERα: Estrogen receptor alpha
IHC: Immunohistochemistry
DAB: Diaminobenzidine
SOD: Superoxide dismutase
TAC: Total antioxidant capacity
HPG: Hypothalamic-pituitary–gonadal
HPA: Hypothalamic–pituitary–adrenal
CRH: Corticotropin-releasing hormone
GnRH: Gonadotropin-releasing hormone
AMH: Anti-Müllerian hormone
